# Tolerability of obinutuzumab therapy in patients with rituximab-relapsed/refractory B-cell malignancies - a retrospective single center cohort study

**DOI:** 10.18632/oncotarget.25714

**Published:** 2018-07-06

**Authors:** Theo Pirich, Elisabeth Zwickl-Traxler, Martin Pecherstorfer, Josef Singer

**Affiliations:** ^1^ Department of Internal Medicine II, University Hospital Krems, Karl Landsteiner University of Health Sciences, Krems an der Donau, Austria

**Keywords:** obinutuzumab, tolerability, rituximab-relapsed/refractory, chronic lymphocytic leukemia, follicular lymphoma

## Abstract

**Background & Aim:**

In randomised clinical trials, the type II anti-CD20 antibody obinutuzumab has been shown to be more effective than rituximab for therapy of chronic lymphocytic leukemia (CLL) and follicular lymphoma (FL). However, this enhanced efficacy was linked with elevated rates of high-grade adverse events. The aim of this study was to assess the tolerability and toxicity profile of obinutuzumab treatment in routine patients with CLL and FL, of whom the majority had experienced toxicity or resistance to rituximab.

**Methods:**

This retrospective cohort study investigated fifteen obinutuzumab-treated patients, eight with CLL and seven with FL. The course of the disease, comorbidities and treatment-related toxicities were recorded. All patients with CLL and all but three FL patients had any form of pre-treatment with rituximab.

**Results:**

Between October 2014 and August 2017, 15 patients were treated with obinutuzumab at the University Hospital Krems. In the CLL-cohort, 1 patient (12,5%) developed pneumonia, 2 (25%) febrile neutropenia, 6 (75%) anemia and 7 (87,5%) thrombocytopenia, respectively. One patient exhibited an infusion-related allergic reaction. In the FL-cohort, 6 patients (85,7%) presented with thrombocytopenia, 3 (42,9%) with anemia and one patient with neutropenia. No sepsis or consecutive solid tumors were seen in any of the patients.

**Conclusion:**

Obinutuzumab was mostly well tolerated in mild to heavily pre-treated patients with CLL and therapy-naïve or pre-treated patients with FL. The frequency and profile of adverse events and toxicity was comparable to data from previous clinical studies and could be managed adequately in the setting of a University Clinic.

## INTRODUCTION

### Chronic lymphocytic leukemia

The WHO-classification (World Health Organisation) describes chronic lymphocytic leukemia (CLL) as an indolent lymphocytic lymphoma, characterized by leukemic progression [1]. It is a B-cell neoplasia and the most common leukemic disease in central Europe [2]. The overall incidence is about 4/100.000 people per year and increases with age. Men develop CLL more frequently than women. The median age at initial diagnosis is 72 years [2, 3]. The majority of patients are asymptomatic at diagnosis. Symptoms can be enlarged and palpable lymph nodes; 10% of patients present with B-symptoms (unexplained fevers, unintentional >10% body weight loss in the preceding 6 months, or drenching night sweats) [4]. Upon progression, lymphadenopathy, spleno- and hepatomegaly as well as signs of bone marrow insufficiency and autoimmune-cytopenia can be present [4].

The clinical staging systems devised by Rai et al. [5] and Binet et al. [6] are regularly applied in clinical routine, as both systems describe major subgroups with discrete clinical outcomes, are simple to apply and are inexpensive [5–8]. The current NCCN guidelines (National Comprehensive Cancer Network, USA, www.nccn.org) suggest that CLL does not require treatment until symptoms are present or disease progression, causing severe cytopenia, can be seen [4, 7]. In clinical practice, patients with asymptomatic early-stage disease (Rai 0, Binet A) should be observed without therapy unless they show signs of disease progression, as in these stages the early use of alkylating agents did not lead to prolonged survival [9]. Patients at intermediate (Rai I and II) and high-risk stages (Rai III and IV) according to the modified Rai classification or at Binet stages B or C usually benefit from the initiation of treatment [7]. The first-line therapy should be chosen based on the cytogenetic status, the comorbidities and the age of the patient. If the del(17p) mutation is not present and the patient is fit and younger than 65 years, immuno-chemotherapy with rituximab, fludarabine and cyclophosphamide should be applied [10]. If therapy-limiting co-morbidities are present, less toxic therapies such as rituximab-bendamustine, ofatumumab, obinutuzumab-chlorambucil or ibrutinib should be chosen [11–14].

If del(17p) or TP53-mutations are present, immuno-chemotherapies show significantly less response-rates and therefore targeted therapies such as ibrutinib (targeting Bruton's tyrosine kinase, Btk) [13–15], or idelalisib (targeting the delta isoform of the phosphoinositide 3-kinase, PIK3CD) should be preferred [16].

### Follicular lymphoma

Follicular lymphoma (FL) is an indolent Non-Hodgkin lymphoma (NHL), characterized by a slowly progressive lymphadenopathy [17]. FL is the second most common lymphoma in the Western World (approximately 35% of NHL-patients and 70% of all indolent lymphomas) [17, 18]. The median age at diagnosis is about 65 years [19]. The clinical course of FL is heterogeneous, ranging from very indolent growth to rapid progression. Asymptomatic lymphadenopathy is one of the leading symptoms in patients with FL at early stages. While bone marrow involvement can be found in up to 70% of patients, other organs are less frequently affected. Patients can present also with B-symptoms or with an increased level of serum lactate dehydrogenase (LDH) [17, 20]. Transformation of FL to a diffuse large B-cell lymphoma (DLBCL) may occur in 10-70% of patients over time (the risk is between 2-3% per year) and is typically accompanied with rapid progression of lymphadenopathy, extra-nodal disease, B-symptoms and elevated serum LDH [17, 21, 22].

The clinical staging is performed by means of the Ann Arbor classification [23]. Various models evaluating the prognosis of FL have been developed. The follicular lymphoma prognostic index (FLIPI) is a simple and reproducible prognostic index, based on easily available clinical data [24]. The FLIPI stratifies patients into 3 risk groups: low (0-1 point), intermediate (2 points) and high (≥ 3 points). In low-risk patients, the 10-year overall survival (OS) is 71%, which indicates that optimal treatment for these patients should avoid toxicity and focus to preserve the quality of life. On the contrary, patients with high-risk follicular lymphoma have a median survival of about 5 years [24]. 10-15% of newly diagnosed patients with follicular lymphoma have early stage disease (Ann Arbor I/II) [18]. In general, radiation therapy should be the treatment strategy for limited stage FL, as it has a curative ambition and displays overall survival rates of 60-80%, with a median survival of about 19 years [25]. Alternatively, watchful waiting or rituximab monotherapy may be considered to avoid the adverse effects of radiation in selected cases [18]. For advanced stage disease (Ann Arbor III/IV) no curative therapy is established yet [18]. Asymptomatic patients do not require immediate treatment unless they have symptomatic nodal or organ enlargement, compromising disease or cytopenia [17], as early initiated chemotherapy or rituximab monotherapy did not show a positive effect with regard to overall survival (OS) [26–28].

The combination of rituximab and chemotherapy resulted in improved overall response rates (ORR), longer progression free survival (PFS) and OS of FL patients compared to treatment with chemotherapy alone [29]. In order to achieve complete remission and long PFS, rituximab in combination with chemotherapy such as CHOP (Cyclophosphamide, doxorubicin (Hydroxydaunorubicin), vincristine (Oncovin^®^) and Prednisone-containing regimen) or bendamustine should be applied [18]. Rituximab maintenance therapy has been demonstrated to improve PFS. This therapy should be given for 2 years [30] as a shorter maintenance period resulted in inferior benefit [31].

### Rituximab

The chimeric, type I anti-CD20 monoclonal antibody (mAb) rituximab (Rituxan^®^, MabThera^®^) is an indispensable cornerstone in treatment algorithms of B-cell malignancies. When combined with chemotherapy, rituximab could significantly improve ORR, PFS and, in some cases, OS of patients with FL, DLBCL, marginal zone lymphoma (MZL), mantle cell lymphoma (MCL), as well as B-CLL [32]. Rituximab was first approved by the US Federal Drug Agency (FDA) in 1997 and in 1998 by the European Medicines Agency (EMA) [32]. It has been approved in 2004 for the first-line treatment of patients with indolent lymphoma in combination with chemotherapy [33].

CD20 is a cell surface protein, expressed almost exclusively on B-cells since pre-B-cell stage but is lost during plasma cell differentiation. CD20 is a mainly intracellularly localized membrane receptor with merely two extracellular loops, the larger of them containing the target epitopes of rituximab and obinutuzumab [34]. In resting B-cells, CD20 is neither shed nor internalized, making it an ideal antigen for targeting with therapeutic antibodies [32].

Rituximab destroys B-cells by multiple mechanisms including complement-dependent cytotoxicity (CDC), antibody-dependent cell-mediated cytotoxicity (ADCC), induction of apoptosis and sensitization to chemotherapy [35]. Main adverse effects include cytokine-mediated reactions, cytopenia, reduced immunity to infection, although in clinical practice sequelae are infrequent [32]. The most frequent infusion related reactions (IRRs) are flu-like syndromes (fever, chills and rigor) [33]. Additional hypersensitivity reactions like tumor lysis syndrome (TLS), can be observed [33]. In summary, rituximab monotherapy as well as applications in combination with chemotherapies (like R-CHOP or R-bendamustine) are generally well tolerated and patients do not experience significant adverse events caused by rituximab [33].

### Obinutuzumab

Obinutuzumab (GA101, branded Gazyva^®^ in USA and Gazyvaro^®^ in Europe) is a novel, type II, glyco-engineered, humanized anti-CD20 monoclonal antibody. It has been developed to enhance efficacy in patients with B-cell malignancies by inducing higher levels of immune cell-mediated tumor cell killing and direct cell death when compared with rituximab [36, 37]. The post-translational glyco-engineering process of this antibody aims to stimulate antibody-dependent cell-mediated cytotoxicity (ADCC) and -phagocytosis (ADCP) by increasing binding affinity to the FcγRIII receptor on immune effector cells [37, 38].

Different forms of “glyco-engineering” exist and are currently heavily tested in cancer research, e.g. the investigational compound ocaratuzumab shows nine amino acid changes in its Fc-region when compared to rituximab. This leads to an up to 20-fold increased CD20 affinity and 6-fold increased ADCC-induction [39]. Several glyco-engineered antibodies have also entered clinical trials, e.g. the anti-IL-5 receptor antibody benralizumab for the treatment of asthma [40]. However, obinutuzumab is up till now the only FDA- or EMA-approved glyco-engineered monoclonal antibody for hemato-oncological indications.

Obinutuzumab has a modified elbow-hinge amino acid sequence, which together with the specific epitope recognized by obinutuzumab results in spatial alterations of the CD20-mAb complex on B-cells when compared to rituximab [34, 38]. This defines obinutuzumab as a type II antibody compared to the type I antibody rituximab [34]. The molecular mechanism of action of obinutuzumab is based on both, its type II character and cell death induction that can be turned on and off by changing this elbow-hinge region [38]. Unlike the inter-tetrameric CD20 binding of type I antibodies (like rituximab), intra-tetrameric binding of type II antibodies does not lead to FcγRIIb-mediated internalization [41].

Goede et al. showed in a large phase III clinical trial, that combining obinutuzumab with chlorambucil improved outcomes in patients with CLL. In this study, obinutuzumab was superior to rituximab by prolonging PFS and achieving higher complete responses as well as molecular responses. However, also infusion-related reactions and neutropenia were more common in the obinutuzumab-chlorambucil group than with rituximab-chlorambucil-treated patients. Despite these higher numbers of neutropenia, the risk of infection though was not increased in the obinutuzumab-treated patient group [12].

Additionally, Sehn et al. could demonstrate that obinutuzumab plus bendamustine followed by obinutuzumab maintenance therapy significantly improved the efficacy of bendamustine monotherapy in rituximab-refractory patients with indolent NHL. As this therapy regimen displayed manageable toxicity in this clinical trial, obinutuzumab bendamustine has been introduced as a new treatment option for patients that relapsed after a rituximab-based immuno-chemotherapy [42].

In 2017, Marcus et al. showed that obinutuzumab-based immuno-chemotherapy and obinutuzumab maintenance therapy resulted in longer progression-free survival than rituximab-based therapy with the same chemotherapy backbone. However, high-grade adverse events were more common with the obinutuzumab-based therapy regimen, confirming the notion that obinutuzumab is more effective but also more hazardous than rituximab. Based on this study, obinutuzumab was approved as a first line treatment of FL [43].

### Research question and aim of the study

The anti-CD20 antibody obinutuzumab has been shown to be more effective than rituximab in treatment-refractory CLL and FL. However, in randomized phase III studies adverse events were more commonly observed and of increased severity than with rituximab-treatment, but not at the expense of a significant reduction of health-related quality of life [12, 42, 44].

We thus aimed to investigate the toxicity of obinutuzumab in a real life patient cohort that has in large part received rituximab treatment and reacted either with adverse events or was relapsed after or refractory to it.

This study assesses the tolerability and toxicity profile of obinutuzumab in patients with CLL and FL, who have been treated at the Internal Medicine Department 2 of the University Hospital Krems.

## RESULTS

Between October 29^th^, 2014 and August 1^st^, 2017, 8 patients with CLL and 7 patients with FL were treated with obinutuzumab at the University Hospital Krems. The general characteristics of the patient cohort are shown in Table 1. Gender distribution was inequal with more male patients treated for CLL and more female patients for FL. The median age at start of obinutuzumab therapy was 68 years, 71 for patients with CLL and 65 for FL patients. The youngest person in the CLL cohort was 56, while the oldest was 81 years old. The age of patients suffering from FL ranged between 51 and 73 years.

**Table 1:** General characteristics of all included patients (n=15)

	CLL	FL
Patients; n (% of all patients)	8 (53,3)	7 (46,7)
Male sex; n (% of all patients)	5 (33,3)	3 (20)
Female sex; n (% of all patients)	3 (20)	4 (26,7)
Age at start of therapy in years; median (min. - max.)	71 (56-81)	65 (51-73)

Abbreviations: CLL: Chronic lymphocytic leukemia; FL: Follicular lymphoma.

As shown in Table 2, all CLL patients were classified according to the two most common CLL staging systems specified by Binet [6] and Rai [5]. Five CLL patients were in stage Binet B and three in Binet C at the start of obinutuzumab therapy. According to the Rai staging system, three patients had Rai I, two had Rai II, one patient Rai III and two patients Rai IV. We also calculated the CLL risk score for all CLL patients. Table 3 shows that 62,5% of the patients were at intermediate and 37,5% at high risk.

**Table 2:** CLL staging according to Binet and Rai classifications

	CLL patients; n (% of all CLL patients)
Binet A	0
Binet B	5 (62,5)
Binet C	3 (37,5)
Rai 1	3 (37,5)
Rai 2	2 (25)
Rai 3	1 (12,5)
Rai 4	2 (25)

**Table 3:** CLL Risk Scores

	CLL patients; n (% of all CLL patients)
CLL Risk Score Low	0
CLL Risk Score Intermediate	5 (62,5)
CLL Risk Score High	3 (37,5)

Tables 4 and 5 give an overview about the FL patient cohort. Table 4 shows the distribution of patients across the different Ann Arbor [23] stages. Four patients had Ann Arbor stage 2 disease, one patient Ann Arbor 3 and two patients were staged Ann Arbor 4. Table 5 displays the distribution of the different FLIPI scores: the majority of patients (n = 4) were FLIPI 2, with one patient each in the FLIPI 1, 3 or 4 groups.

**Table 4:** FL staging with Ann Arbor classification

	FL patients; n (% of all FL patients)
Ann Arbor 1	0
Ann Arbor 2	4 (57,1)
Ann Arbor 3	1 (14,3)
Ann Arbor 4	2 (28,6)

**Table 5:** FLIPI scores of FL patients

	FL patients; n (% of all FL patients)
FLIPI 1	1 (14,3)
FLIPI 2	4 (57,1)
FLIPI 3	1 (14,3)
FLIPI 4	1 (14,3)

Abbreviations: FLIPI: Follicular Lymphoma Prognostic Index.

The number and type of pre-treatments are presented in Table 6. Five patients of the CLL group had received one pre-treatment, one patient 2 pre-treatments and two patients had received 3 pre-treatments each. All patients of the CLL cohort had received rituximab in combination with either bendamustine (n = 7) or fludarabine/cyclophosphamide (n = 2) prior to obinutuzumab treatment. Three patients in the FL group had not received any pre-treatment, while one patient had received 1 pre-treatment, two 2 pre-treatments and one patient 4 pre-treatments. In comparison to the CLL group only 57,1% of the FL cohort had a form of rituximab pre-treatment either in combination with CHOP (n = 2) or with bendamustine (n = 2). No stem cell transplantation was performed in any of the patients, neither of the CLL nor the FL cohort.

**Table 6:** Pre-treatments

	CLL; n (% of all CLL patients)	FL; n (% of all FL patients)
No pre-treatment	0	3 (42,9)
1 pre-treatment	5 (62,5)	1 (14,3)
2 pre-treatments	1 (12,5)	2 (28,6)
3 pre-treatments	2 (25)	0
4 pre-treatments	0	1 (14,3)
R-Bendamustine	7 (87,5)	2 (28,6)
R-CHOP	0	2 (28,6)
CHOP	0	2 (28,6)
Rituximab (in combination with either CHOP, bendamustine or fludarabine/cyclophosphamide)	8 (100)	4 (57,1)
Stem cell transplantation	0	0

Abbreviations: CHOP: Cyclophosphamide, doxorubicin (Hydroxydaunorubicin), vincristine (Oncovin^®^) and Prednisone-containing chemotherapy regimen.

As shown in Table 7, one patient from each disease cohort has been suffering from atrial fibrillation. In the CLL group, two patients had coronary artery disease or diabetes mellitus type II as comorbidities. One patient of the FL group had a history of DLBCL and eradicated hepatitis C.

**Table 7:** Comorbidities

	CLL; n (% of all CLL patients)	FL; n (% of all FL patients)
Atrial fibrillation	1 (12,5)	1 (14,3)
Coronary artery disease	2 (25)	0
Diabetes mellitus type II	2 (25)	0
Hematologic comorbidities	0	1 previous DLBCL (14,3)
Hepatitis C	0	1 eradicated Hepatitis C (14,3)

Abbreviations: DLBCL: Diffuse Large B-cell Lymphoma.

**Figure 1:**
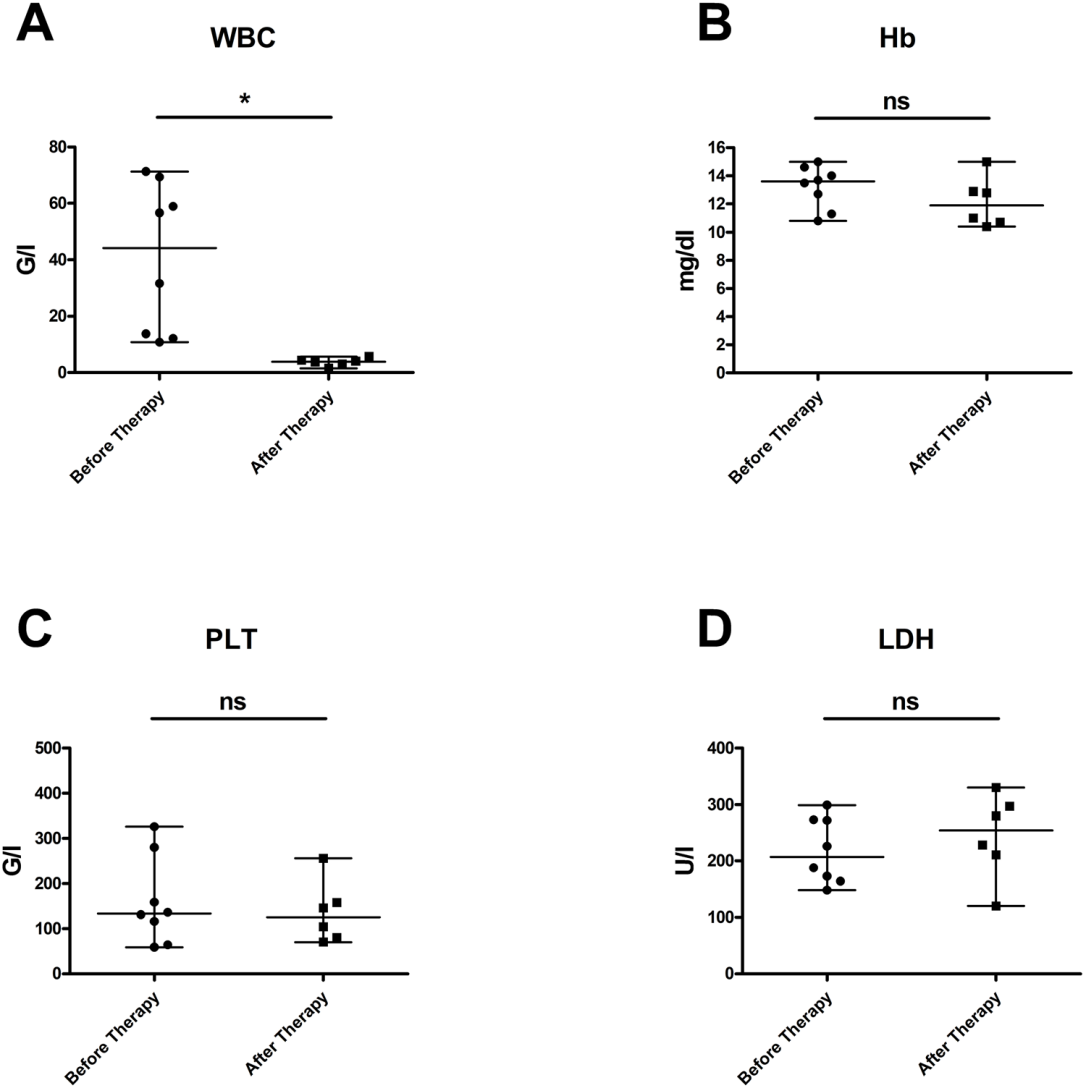
Laboratory values in patients with CLL at start of obinutuzumab therapy and after immuno-chemotherapy with obinutuzumab-bendamustine **(A)** demonstrates that the elevated leukocyte counts could be effectively reduced (p = 0,0169); **(B)** hemoglobin values (p = 0,3322), **(C)** platelet counts (p = 1,0) and **(D)** LDH levels (p = 0,4378) were not significantly altered. Median values are displayed, whiskers indicate maximum and minimum values.

In Figure 1, laboratory values of CLL patients at the start of obinutuzumab therapy and after immuno-chemotherapy with obinutuzumab-bendamustine are illustrated: Panel A demonstrates the leukocyte count (WBC), B hemoglobin values (Hb), C platelet count (PLT) and in D lactate dehydrogenase (LDH) levels are displayed. As expected, immuno-chemotherapy with obinutuzumab-bendamustine could significantly reduce the elevated numbers of leukocytes in peripheral blood (p=0.0169), whereas hemoglobin values, platelet counts and LDH levels were not significantly altered.

**Figure 2:**
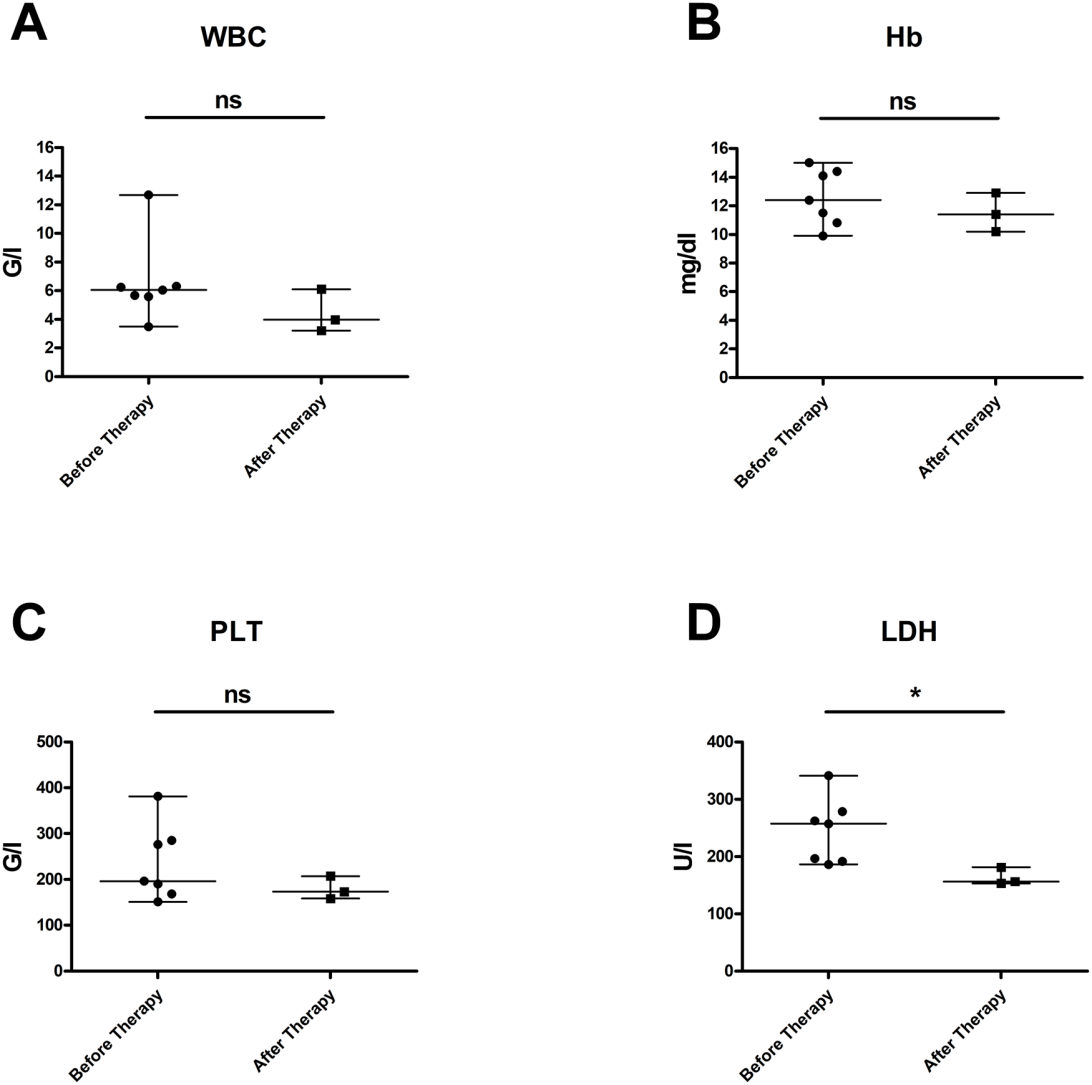
Laboratory values in patients with FL at start of obinutuzumab therapy and after immuno-chemotherapy with obinutuzumab-bendamustine **(A)** Leukocyte counts (p = 0,4985), **(B)** hemoglobin values (p = 0,2153) and **(C)** platelet counts (p = 0,5144) were not significantly altered; **(D)** elevated LDH levels could be effectively reduced (p = 0,0144). Median values are displayed, whiskers indicate maximum and minimum values.

Figure 2 displays the laboratory values of FL patients at the start of obinutuzumab therapy and after immuno-chemotherapy with obinutuzumab-bendamustine: here, increased LDH levels could be significantly reduced (p=0.0144), whereas WBC, Hb and PLT levels were not significantly affected.

Incidences of adverse events occurring during obinutuzumab-bendamustine therapy are shown in Table 8. These adverse events were staged according to the Common Terminology Criteria for Adverse Events Version 5.0 (CTCAEv5.0) of the National Cancer Institute (NCI) of the USA. In the CLL cohort, 12,5% of the patients experienced pneumonia, 25% febrile neutropenia, 75% anemia and 87,5% thrombocytopenia, respectively. In general, these adverse events were of low grades, with only one grade 4 thrombocytopenia and one grade 4 febrile neutropenia. One patient had an infusion-related allergic reaction (grade 3). In the FL cohort, 85,7% of the patients experienced thrombocytopenia, 42,9% anemia and one patient neutropenia, respectively. Again here, these adverse effects were generally of lower grades, with only one grade 3 thrombocytopenia and one grade 4 neutropenia.

**Table 8:** Adverse effects during obinutuzumab-bendamustine therapy

	CLL; n (% of all CLL patients)						FL; n (% of all FL patients)					
	total	CTCAE Grade 1	2	3	4	5	total	CTCAE Grade 1	2	3	4	5
Infusion-related allergic reactions	1 (12,5)	0	0	1 (12,5)	0	0	0	0	0	0	0	0
Anemia	6 (75)	3 (37,5)	1 (12,5)	1 (12,5)	0	0	3 (42,9)	3 (42,9)	0	0	0	0
Thrombocytopenia	7 (87,5)	4 (50)	1 (12,5)	1 (12,5)	1 (12,5)	0	6 (85,7)	5 (71,4)	0	1 (14,3)	0	0
Leukopenia	4 (50)	3 (37,5)	1 (12,5)	0	0	0	6 (85,7)	5 (71,4)	0	1 (14,3)	0	0
Neutropenia	2 (25)	0	0	1 (12,5)	1 (12,5)	0	2 (28,6)	1 (14,3)	0	0	1 (14,3)	0
Febrile Neutropenia	2 (25)	0	0	1 (12,5)	1 (12,5)	0	0	0	0	0	0	0
Pneumonia	1 (12,5)	0	0	1 (12,5)	0	0	0	0	0	0	0	0
Bronchial Infection	1 (12,5)	0	1 (12,5)	0	0	0	0	0	0	0	0	0
Diarrhea	1 (12,5)	0	1 (12,5)	0	0	0	0	0	0	0	0	0
Sepsis	0	0	0	0	0	0	0	0	0	0	0	0
Solid tumors	0	0	0	0	0	0	0	0	0	0	0	0

Abbreviations: CTCAE: Common Terminology Criteria for Adverse Events.

**Figure 3:**
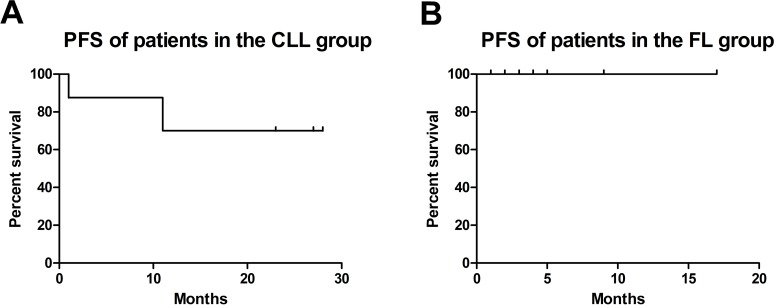
Progression-free survival (PFS) of patients in the CLL **(A)** and FL **(B)** group.

No sepsis or subsequent solid tumors were experienced in any of the patients, neither in the CLL nor FL cohort. In the CLL group, two patients required red blood cell transfusion and one patient thrombocyte substitution. Table 9 provides information about adverse effects that developed after therapy: one patient of the CLL cohort experienced a neuroborreliosis 5 months after the last obinutuzumab-bendamustine cycle. Figure 3 shows progression-free survival (PFS) of patients in the CLL group (A) and in the FL group (B). Two out of eight patients of the CLL group died: one female patient after one cycle of obinutuzumab-bendamustine. She developed persistent and therapy-refractory leukopenia, exsiccosis and cardiac arrest. Here, a secondary hematologic malignancy could be unveiled, a Multiple Myeloma. The other, male patient died due to pneumonia after having completed 6 cycles of obinutuzumab-bendamustine. Here, after a short initially good response, recurrence of the disease with a CD20 negative clone occurred. Five out of eight CLL patients completed all scheduled cycles of obinutuzumab-bendamustine. Two patients were still on therapy as of August 1^st^, 2017. All patients were still alive in the FL group. Three out of seven FL patients completed all scheduled cycles of obinutuzumab-bendamustine. Four patients were still on therapy as of August 1^st^, 2017.

**Table 9:** Adverse effects after obinutuzumab-bendamustine therapy until the end of the observation period

	CLL	FL
Neuroborreliosis	1 (12,5)	0

## DISCUSSION

This retrospective, single-center study analyzed the tolerability and toxicity of obinutuzumab in patients with CLL or FL. Between October 29^th^, 2014 and August 1^st^, 2017, 15 patients with CLL (n = 8) or FL (n = 7) were treated with obinutuzumab-bendamustine and followed up at the University Hospital Krems. In our CLL patient cohort, gender distribution was slightly shifted towards male sex, while in the FL subgroup female sex was more frequent. The median age at start of obinutuzumab therapy was 68: in detail 71 years for patients with CLL and 65 years for patients with FL, which is very similar to other published patient cohorts [12, 42]. We also calculated the CLL risk score for all CLL patients: 62,5% of the patients were at intermediate risk and 37,5% at high risk. The fact that in our study no patients with a low risk profile were treated is in agreement with the finding of a meta-analysis of 6 clinical trials in patients with early-stage disease demonstrating that the use of early treatment initiation did not lead to prolonged survival in comparison to a “watch and wait strategy” [9]. It is notable that both our patient cohorts had relevant clinical comorbidities, mainly atrial fibrillation (n = 2), coronary artery disease (n = 2) or diabetes mellitus type II. In the FL group one patient had a history of DLBCL and eradicated hepatitis C. Although also “frail” patients were included in our study cohort, the rate of adverse events was acceptable and manageable in the context of a University Clinic.

Previous randomized clinical trials evaluating the efficacy and safety of obinutuzumab in NHL were performed in both untreated [43] and pre-treated [42] patients refractory to standard therapy. In both phase III trials, obinutuzumab was well tolerated [42, 43]. In fact, all but three of our patients in the FL group and all patients in the CLL cohort had undergone at least one pre-treatment, in some cases multiple therapies. Rituximab has been defined as standard of care in both FL and CLL. All patients of the CLL cohort received rituximab in combination with either bendamustine (n = 7) or fludarabine/cyclophosphamide (n = 2). In comparison to the CLL group only 57,1% of the FL cohort had any form of rituximab pre-treatment, here either in combination with CHOP (n = 2) or bendamustine (n = 2). This is due to the fact, that obinutuzumab received EMA-approval also for first line therapy of FL during our observation period and was thus implemented as first line therapy in the clinical routine of the University Hospital Krems. Although this patient cohort of untreated FL patients is with n = 3 rather small, no significant differences could be observed in the tolerability of obinutuzumab compared to pre-treated patients. However, this has to be further investigated in long-term clinical observation studies.

Preterm termination of rituximab therapy might be necessary in some cases. Four out of eight (50%) CLL patients of our cohort had terminated rituximab therapy early because two of them showed an infusion-related allergic reaction. Of note, this small group of patients tolerated obinutuzumab without evidence of an infusion-related allergic event and both completed the treatment regimen as prescribed. Of the remaining two patients who had stopped rituximab, one female patient developed thrombocytopenia, the other (male) patient experienced anemia as well as thrombocytopenia while obinutuzumab was applied. Both, thrombocytopenia and anemia were also commonly reported in previous clinical trials [12, 42]. Infusion-related allergic reactions, neutropenia, thrombocytopenia, anemia, pyrexia, cough and musculoskeletal disorders were among the most commonly observed adverse reactions when obinutuzumab was combined with chlorambucil in CLL [45]. In our observational study one patient with CLL experienced an infusion-related allergic reaction at the first cycle. Goede et al. observed that infusion-related reactions occurred in approximately 20% of patients during the first infusion of obinutuzumab [12]. Because of this high rate of infusion-related reactions, the first dose of obinutuzumab should be split between day 1 (100 mg) and day 2 (900 mg) when applied to CLL patients [45]. We strictly applied that protocol also for our FL patients, which may be one factor, why our rate of infusion-related reactions was that low.

In the FL group, four out of seven (57,1%) patients had received rituximab. One of them had stopped rituximab therapy early because of an allergic reaction but tolerated obinutuzumab well. However, this patient developed anemia and thrombocytopenia during obinutuzumab therapy. Sehn et al. found that obinutuzumab plus bendamustine followed by obinutuzumab maintenance therapy in rituximab-refractory patients with indolent NHL resulted in serious adverse events in 38% and death due to adverse events in 6% of the patients, respectively. Neutropenia (33%), thrombocytopenia (11%), anaemia (8%) and infusion-related reactions (11%) were observed in this trial [42]. Therefore, patients should be regularly monitored, also during the maintenance period.

None of our patients underwent stem cell transplantation, which is partly due to the fact, that stem cell transplantations are not performed at the University Hospital Krems, and thus patients are referred to and treated at other hospitals. However, one male patient was offered stem cell transplantation, but refused out of personal reasons. Two out of 15 patients died during or after completing the therapy with obinutuzumab. These deaths were not therapy-related, as one had a second haematological malignancy, a Multiple Myeloma, which was unveiled during the obinutuzumab-bendamustine therapy. The other patient developed after an initially good response an early relapse with a CD20-negative CLL-clone.

Our findings are in agreement with large controlled trials showing that obinutuzumab is effective in both patients with CLL or FL but without significant impairment of healthy-related quality of life indices [44]. As this study only examined one centre, potential biases are the small sample size and limited ethnic diversity. Additionally, the patient population is rather rural; there should no difference to be expected in comparison to an urban population, but as this was not investigated in large trials, this could nonetheless be a potential bias.

In conclusion, obinutuzumab was mostly well-tolerated in a group of mild to heavily pre-treated patients with CLL and therapy-naïve or pre-treated patients with FL. Therefore, the planned obinutuzumab-bendamustine regimen could be completed in the majority of patients offering an effective therapeutic alternative to patients refractory to standard therapies. The frequency and profile of adverse events and toxicity was comparable to data from previous clinical studies and could be managed adequately in the clinical setting of a University Clinic. Nonetheless, further studies are needed to characterize the risk of severe adverse events due to obinutuzumab therapy and their appropriate management.

## MATERIALS AND METHODS

The design of this study is an uncontrolled retrospective cohort study using data from the database of the University Hospital Krems: the study-participants were pseudonymised and got a consecutive serial number (001 - 002 - …). The analysis of the study-oriented data took place only with those assigned serial numbers. The following parameters were recorded and used for statistical evaluation:

- Demographic data.
  - Age (at the start of obinutuzumab-bendamustine therapy).
  - Gender.
- Disease-specific parameters.
  - Patient characteristics.
    - Stage of the disease.
    - Risk score.
    - Progression-free survival (PFS).
    - Pre-treatment (type and quantity of pre-treatments).
    - Stem cell-transplantation.
    - Comorbidities.
  - Patient's adverse effects (staged according to the to the Common Terminology Criteria for Adverse Events Version 5.0 (CTCAE v5.0) of the National Cancer Institute (NCI) of the USA):
    - Infectious diseases, pneumonia, sepsis, tumour lysis syndrome, allergic reactions.
    - Need for erythrocyte transfusion, thrombocyte transfusion.
    - Laboratory parameters (WBC, Hb, PLT, LDH).
    - Leukopenia, neutropenia, febrile neutropenia, anaemia, thrombocytopenia.

The registry was approved by the ethics committee of the Karl Landsteiner Private University in June 2017.

### Study population

Participants of the study were patients with CLL (n = 8) and FL (n = 7), who were treated with obinutuzumab-bendamustine after a rituximab-containing therapy in the period of 2014 to 2017 at the Department of Internal Medicine 2 of the University Hospital Krems. The sample size of the study was 15 participants. Exclusion criterion was age below 18 years.

### Treatment regimen

All patients received immuno-chemotherapy with obinutuzumab and bendamustine for 6 cycles at intervals of 28 days. During each cycle, patients received 1000 mg obinutuzumab at day 1 and 90 mg bendamustine /m^2^ body surface area at days 1 and 2. During the first cycle, obinutuzumab was administered in two doses: 100 mg at day 1, and 900 mg at day 2 plus 1000 mg at days 8 and 15.

### Statistical analysis

The collected data was descriptively analysed using Microsoft Excel (Version 16.9), Microsoft Word (Version 16.9.1) and GraphPad Prism (Version 7.0d). Initially, descriptive analyses of gender and age were performed. Subsequently, laboratory values before and after obinutuzumab therapy were collected and median as well as minimum and maximum values were analysed. Scatter dot plots were chosen to demonstrate the range between minimum and maximum values of leukocyte counts (WBC), haemoglobin values (Hb), thrombocyte counts (PLT) and lactate dehydrogenase levels (LDH). Laboratory values after therapy were determined at the first control visit at our outpatient clinic after having completed immuno-chemotherapy with obinutuzumab-bendamustine. This means that these values are in the FL group before the start of obinutuzumab maintenance therapy. Respective laboratory values before and after therapy were analysed by means of paired t-test, and statistical significance was accepted at *p<0,05*.

### Declaration

This work is based on the bachelor's thesis of Theo Pirich, submitted in May 2018 at the Karl Landsteiner University of Health Sciences entitled “Tolerability of obinutuzumab therapy in patients with rituximab-relapsed/refractory B-cell malignancies - a retrospective uncontrolled cohort study”.

## Abbreviations

ADCC: Antibody-dependent cell-mediated cytotoxicity
ADCP: Antibody-dependent cell-mediated phagocytosis
CDC: Complement-dependent cytotoxicity
CHOP: Cyclophosphamide, doxorubicin (Hydroxydaunorubicin), vincristine (Oncovin^®^) and Prednisone-containing chemotherapy regimen
CLL: Chronic lymphocytic leukemia
CTCAE: Common Terminology Criteria for Adverse Events
DLBCL: Diffuse large B-cell lymphoma, e.g. exempli gratia (for example)
FL: Follicular lymphoma
FLIPI: Follicular Lymphoma Prognostic Index
Hb: Hemoglobin, i.e. id est (that is)
IRR: Infusion related reaction
LDH: Lactate dehydrogenase
mAb: Monoclonal Antibody
MBL: Monoclonal B-lymphocytosis
n: number
NCCN: National Comprehensive Cancer Network
NCI: National Cancer Institute
NHL: Non-Hodgkin lymphoma
OS: Overall survival
ORR: Overall Response Rate
PFS: Progression-free survival
PLT: Platelets
RR: Response Rate
TLS: Tumor lysis syndrome
WBC: White blood cells
WHO: World Health Organization.
